# Damage Resistance of an fMRI-Spiking Neural Network Based on Speech Recognition Against Stochastic Attack

**DOI:** 10.3390/biomimetics10070415

**Published:** 2025-06-26

**Authors:** Lei Guo, Huan Liu, Yihua Song, Nancheng Ma

**Affiliations:** 1 Tianjin Key Laboratory of Bioelectromagnetic Technology and Intelligent Health, School of Health Sciences and Biomedical Engineering, Hebei University of Technology, Tianjin 300131, China; 202011402002@stu.hebut.edu.cn (H.L.); 202212901005@stu.hebut.edu.cn (Y.S.); 202322902019@stu.hebut.edu.cn (N.M.); 2 State Key Laboratory of Reliability and Intelligence of Electrical Equipment, Hebei University of Technology, Tianjin 300131, China

**Keywords:** spiking neural network, functional magnetic resonance imaging, damage resistance, speech recognition

## Abstract

Brain-like models are commonly used for pattern recognition, but they face significant performance degradation in neuromorphic hardware when exposed to complex electromagnetic environments. The human brain has adaptability to the exterior attack, and we expect that incorporating bio-plausibility into a brain-like model will enhance its robustness. However, brain-like models currently lack bio-plausibility. Therefore, we construct a spiking neural network (SNN) whose topology is constrained by human brain functional Magnetic Resonance Imaging (fMRI), called fMRI-SNN. To certify its damage resistance, we investigate speech recognition accuracy against stochastic attack. To reveal its damage-resistant mechanism, we explore the neural electrical features, adaptive modulation of synaptic plasticity, and topological features against stochastic attack. Research shows that fMRI-SNN surpasses SNNs with distinct topologies in recognition accuracy against stochastic attack, notably maintaining similar accuracy levels before and after stochastic attacks when the damage proportion is below 30%, demonstrating that our method improves the damage resistance of brain-like models. In addition, the change in neural electrical activity serves as interior manifestation, corresponding to the damage resistance of SNNs for recognition tasks, while the synaptic plasticity serves as the inherent determinant of the damage resistance, and the topology serves as a determinant impacting the damage resistance.

## 1. Introduction

Brain-like neuromorphic hardware is commonly used for pattern recognition tasks, and has demonstrated a strong performance in speech recognition, image classification, and obstacle avoidance. Wu et al. [1] implemented a spiking neural network (SNN) with dynamic memristor-based time-surface neurons on complementary metal–oxide semiconductor (CMOS) hardware network architecture, and applied it in a speech recognition task on the Heidelberg digits dataset. They found that the speech recognition accuracy of this SNN hardware is 95.91%. Han et al. [2] implemented SNN on Field-Programmable Gate Arrays (FPGA), and applied it in a handwritten digits classification task on the MNIST dataset. They found that the classification accuracy of this SNN hardware is 97.06%, and the power consumption is only 0.477 W. However, electromagnetic interference can damage the components of electronic equipment, resulting in a decline or even failure of their normal function [3]. As a product of natural selection, the human brain is adaptive to exterior attack [4]. Thus, a brain-like model, by incorporating self-adaptability, could be expected to improve its robustness; however, existing models are limited by their lack of bio-plausibility. As a brain-like model, the SNN contains neuron models, synaptic plasticity models, and topology, which offers a powerful processing capability for temporal-related signals through simulating electrophysiological properties [5].

Neurons are the fundamental structural and functional units of the brain, and neuron models have been proposed to simulate the electrophysiological characteristics of neurons based on mathematical equations. As a first-order ordinary differential equation (ODE), the integrate-and-fire (LIF) neuron model can reproduce the integration and firing process of neuronal membrane potential (MP), which has a low computing cost, but cannot conform well to the firing characteristics of the neuron, resulting in a lack of bio-reality [6]. Due to the simplicity of the LIF model, Rhodes et al. [7] employed the LIF model to construct an SNN on the SpiNNaker neuromorphic hardware platform, and measured its computing speed and energy efficiency. They found that the computing speed and energy efficiency of this neuromorphic hardware, with parallelized spike communication, are higher than those of the neuromorphic hardware without parallelized spike communication. Conversely, the Hodgkin–Huxley (HH) neuron model conforms well to the firing characteristics of the neuron, but its fourth-order ODE imposes a high computing cost [8]. Ward et al. [9], respectively, the employed HH model and LIF model to construct SNNs on the SpiNNaker neuromorphic hardware platform, and compared their computing time and firing behaviors. They found that, compared to neuromorphic hardware with the LIF model, this neuromorphic hardware with the HH model has a greater computation time and more diverse firing behaviors. These results verify that neuromorphic hardware with the LIF model has lower bio-reality, and neuromorphic hardware with the HH model has a greater computation cost. The Izhikevich neuron model, as a second-order ODE [10], conforms well to the firing characteristics of the neuron while maintaining a low computing cost, and can be broadly applied in the construction of a brain-like model. Faci-Lázaro et al. [11] built an SNN incorporating the Izhikevich model and investigated the network’s spontaneous activation, which was influenced by both dynamic and topological factors. Their research demonstrated that higher connectivity, shorter internodal distances, and greater clustering collectively reduce the synaptic strength that is necessary to sustain network activity. Li et al. [12] employed the Izhikevich model to construct an SNN in a RRAM-based neuromorphic chip, and investigated its signal transmission. They found that the spike signals generated by the Izhikevich model have rich firing patterns and a high computation efficiency, which enable it to maintain encoding efficiency, even under exterior noise, thereby reducing the signal distortion of the chip. These results demonstrate that the brain-like model, combined with the Izhikevich model, has the advantages of higher bio-reality and a lower computation cost.

Synapses are important structures for information transfer between neurons, and serve as the foundation for learning and memory [13]. The results of biological experiments indicated that excitatory synapses (ES) and inhibitory synapses (IS) jointly form the basis of dynamic modulation in the brain [14]. Drawing on these findings, scholars have incorporated synaptic plasticity models with ES and IS into SNNs. For example, Sun and Si [15] found that an SNN with the synaptic plasticity models co-modulated by ES and IS has higher coding efficiency than the SNN modulated only by ES or IS during the population coding. In synapses, the neurotransmitters’ diffusion across the synaptic cleft induces synaptic time delay (STD), which augments the processing of neural signals. The dynamic range of bio-STD is not fixed, but rather exhibits a stochastic distribution, varying randomly between 0.1 and 40 ms [16]. Zhang et al. [17] employed STD to SNN, and discovered that the learning performance of this SNN surpassed those of SNNs without STD. However, its STD was a fixed value, which did not conform to the bio-STD. The variability in STD is crucial for the modulation of the signal transfer, allowing for an adaptable response to attack. We therefore constructed a complex SNN based on synaptic plasticity models with stochastic STD distributed between 0.1 and 40 ms, conforming to the bio-STD, and discovered that the damage resistance of this SNN was superior to the SNN with fixed STD. In turn, the SNN with a fixed STD was superior to the SNN without an STD against stochastic attack [18]. Therefore, a synaptic plasticity model incorporating STD aligns with the bio-STD, is co-modulated by ES and IS, and can enhance the information processing of an SNN.

The topology reflects the connections that form among neurons, and influences the brain function. Van et al. [19] obtained functional brain networks (FBNs), utilizing resting-state fMRI of healthy humans, and discovered that the networks possessed the small-world (SW) and scale-free (SF) features. The SW network achieves an optimal balance between high clustering coefficients and low path lengths, supporting efficient distributed signal processing capabilities. In contrast, the SF network exhibits a significant node connectivity heterogeneity by following a power–law degree distribution, and this topology utilizes hub node-mediated routing mechanisms to significantly improve information throughput efficiency. Drawing on these findings, scholars have constructed brain-like models with SW or SF features. For examples, Budzinski et al. [20] constructed a small-world spiking neural network (SWSNN) using the Watts–Strogatz (WS) algorithm, and discovered that when the coupling strength was sufficiently high, this SWSNN transitioned from an asynchronous state to an synchronous state as the reconnection probability increased; Reis et al. [21] built a scale-free spiking neural network (SFSNN), employing the Barabasi–Albert (BA) algorithm, and discovered that this SFSNN could inhibit burst synchronization caused by light pulse perturbation, and keep low synchronization when the perturbation stopped. However, the real functional connections of the human brain cannot be reflected by the topology created by algorithms. Due to natural selection and evolution, the human brain has developed efficient computing power [22]. SNNs constrained by the FBN for the human brain have the potential to improve neural information processing.

An SNN possesses the capability to capture and transmit temporal signals through its spiking coding, which makes it superior in handling the temporal-related tasks [23,24]. The speech signals inherently exhibit prominent temporal characteristics, as the rhythm of phonemes, syllables, and words evolves over time. Thus, SR extends beyond the instantaneous classification of acoustic signals, encompassing the continuous monitoring and analysis of temporal dynamics in speech features across an extended time sequence [25]. A liquid state machine (LSM) serves as a framework for recognition tasks, where the reserve layer formed an SNN [26]. This SNN is responsible for transforming the input data from its original lower-dimensional space into a higher-dimensional space, where distinct categories tend to become more recognizable. Additionally, the independence of the reserve layer and reading function in the LSM allows for easier training of the reading function rather than the entire network [27]. Hence, the LSM has been applied to the task of SR. Zhang et al. [28] built an LSM utilizing recurrent SNN with a synaptic plasticity that conformed to Hebbian theory, and observed that its SR outperformed a recurrent SNN with a synaptic plasticity of long short-term memory on the TI-46 dataset. Deckers et al. [29] built an LSM co-modulated by ES and IS, and observed that its SR accuracy outperformed the SNN modulated by the ES on the TI-46 speech corpus. Moreover, the performance of an SNN is influenced by its topology. Srinivasan et al. [30] built an LSM with topology featuring a distributed multi-liquid topology, and observed that its performance outperformed the SNN with a random topology on the TI-46 speech corpus. In our previous research [31,32], we constructed a brain-like model called fMRI-SNN, whose topology was constrained by human brain fMRI data, and discovered that its SR accuracy outperformed that of SNNs with distinct topologies on the TI-46 speech corpus. These findings suggest that improving the bio-plausibility of SNN can enhance its performance, and SR tasks serve as an effective benchmark for certifying the performance of SNN.

Research in the field of biological neuroscience has shown that the human brain is robust to injury [33]. Drawing on these findings, the robustness of brain-like models, including interference resistance and damage resistance, has been investigated. In terms of interference resistance, Zhang et al. [34] found that a novel hybrid residual SNN could eliminate exterior electric-field noise by controlling the pulse frequency, with a recognition accuracy that was only 2.15% lower than that of an SNN without exterior electric-field noise on the neuromorphic DVS128 Gesture dataset. In our previous work [35], we found that, against pulse noise, the SWSNN exhibited superior interference resistance compared to an SFSNN by analyzing the neural electrical activity. In terms of damage resistance, Jang et al. [36] found that against stochastic attacks, a deep neural network incorporating adaptable activation functions had a superior recognition performance to those networks without these same functions on the CIFAR-10 datasets. In our previous work [37], we found that against a targeted attack, the SWSNN exhibited superior damage resistance compared to SFSNN by analyzing the neural electrical activity. Nevertheless, the existing brain-like models currently lack bio-plausibility, and researching a brain-like model with bio-plausibility is expected to improve its robustness. The aim of this study is to construct a brain-like model with bio-plausibility to optimize its damage resistance. We construct an fMRI-SNN constrained by human FBN from fMRI data, then certify its damage resistance on the task of SR, and further investigate the damage-resistant mechanism.

The core contributions of this research are highlighted below:

- We proposed an fMRI-SNN whose topology is constrained by human brain fMRI to improve its bio-plausibility.
- We investigated the SR accuracy of fMRI-SNN against stochastic attack to certify the damage resistance, and found that fMRI-SNN surpasses SNNs with distinct topologies in SR accuracy, notably maintaining similar accuracy levels before and after stochastic attack at the damage proportion below 30%.
- Furthermore, we discussed the damage-resistant mechanism of fMRI-SNN, and found that the change in neural electrical activity serves as an interior manifestation corresponding to the damage resistance of SNNs for recognition tasks, the synaptic plasticity serves as the inherent determinant of the damage resistance, and the topology serves as a determinant impacting the damage resistance.

The structure of this paper is as follows: Section 2 presents the method applied to build the fMRI-SNN; Section 3 certifies the damage resistance of the fMRI-SNN by speech recognition; Section 4 discusses the damage-resistant mechanism of the fMRI-SNN; finally, Section 5 concludes the paper.

## 2. Materials and Methods

In this section, the construction of the SNNs and the SR framework that incorporated an SNN are described.

### 2.1. Construction of SNNs

To increase the bio-plausibility of brain-like models, we construct an fMRI-SNN. To assess the role of network topology in the damage resistance of an SNN, we construct topologically distinct SNNs (SWSNN and SFSNN) for comparison.

#### 2.1.1. Construction of fMRI-SNN

**Figure 1:**
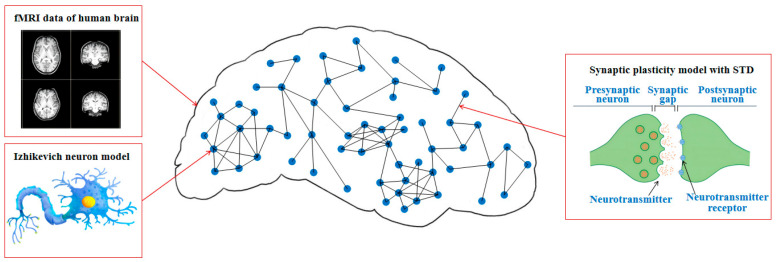
Schematic diagram of fMRI-SNN.

We construct an fMRI-SNN, in which the topology is constrained by an FBN from human fMRI data, the nodes are represented as Izhikevich neuron models, and the edges are represented as synaptic plasticity models with STD. The schematic diagram of fMRI-SNN is presented in Figure 1.

1. Topology

The topology of our fMRI-SNN is derived from FBN, constructed using fMRI data from five subjects (2 males, 3 females; mean age 22.8 ± 0.62). The data are sourced from the publicly available NeuroImaging Tools and Resources Collaboratory (http://www.NITRC.org/projects/fcon_1000, accessed on 23 June 2025.). Brain regions were regarded as nodes in an FBN, and the functional connectivity (FC) among these nodes was used to denote the edges, where the strength of the FC determined the weight of each edge [38].

**Figure 2:**
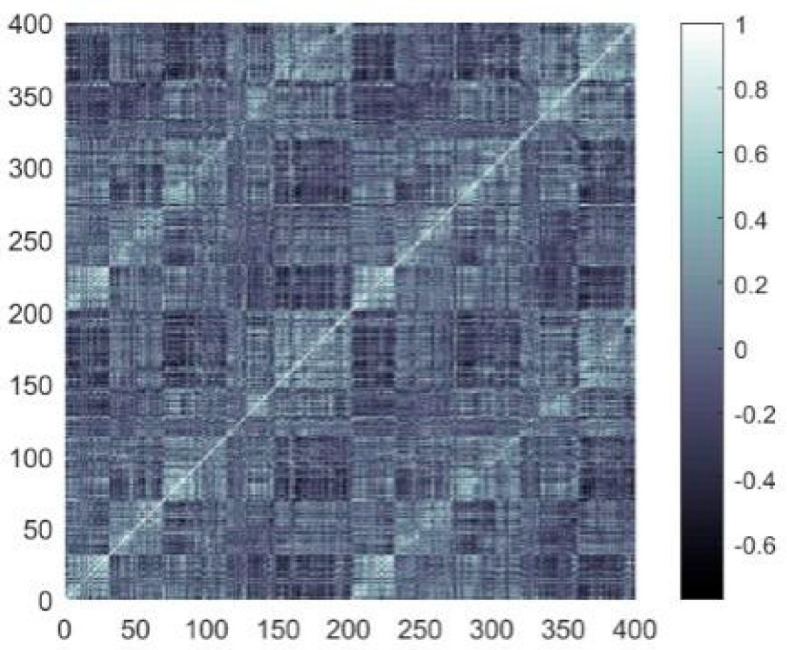
Coefficient matrix (in the color bar, an R value approaching ±1 reflects a stronger link between two network nodes, whereas values near 0 suggest a weaker link).

(1) Network nodes: We employed anatomical automatic labeling (AAL) [39] to segment the brain regions in the fMRI data. This alignment with the AAL atlas resulted in the identification of 400 brain regions, each serving as a network node.

(2) Network edges: The Pearson correlation was employed to compute the strength of the FC among nodes, and the correlation coefficient R was expressed as follows:

(1) $$R=\frac{\sum_{i=1}^{n}\left(A_{i}-\bar{A})(B_{i}-\bar{B}\right)}{\sqrt{\sum_{i=1}^{n}(A_{i}-\bar{A}{)}^{2}}\sqrt{\sum_{i=1}^{n}(B_{i}-\bar{B}{)}^{2}}}$$

where n is the length for the time series of the blood oxygen level-dependent (BOLD) signals; and A and B denote the temporal sequences BOLD signals of distinct nodes. The coefficient matrix produced by computing the Pearson correlation coefficients is presented in Figure 2.

Neuroscience researchers have discovered that the FBN for the human brain exhibits sparsity, with its connectivity density spanning from 3.6% to 39.3% [40,41], and its mean node degree exceeds $2\ln\left(M\right)$ [42], where *M* is the quantity of network nodes. To obtain a topology for our fMRI-SNN that was consistent with the features of an FBN for the human brain, we needed to find an appropriate threshold $X_{th}$. An edge between nodes is created if the absolute strength of FC exceeds $X_{th}$. The mean and standard deviations (SD) of the network density and mean node degree were computed for five subjects across a range of $X_{th}\;$ from 0 to 0.9 in steps of 0.1, as presented in Table 1.

**Figure 3:**
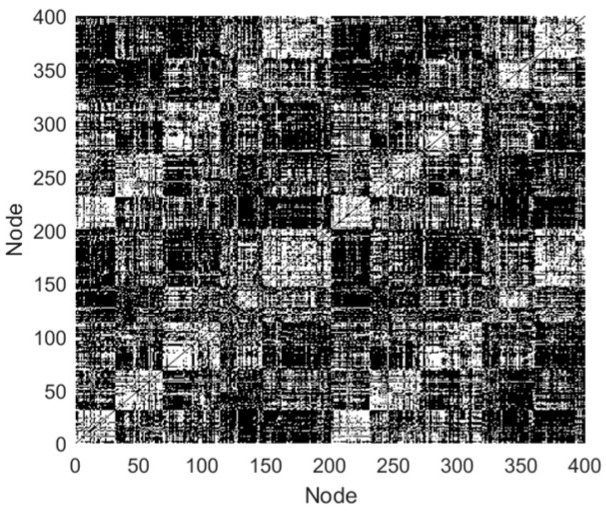
Edge links of the FBN (black indicates functional links between nodes, while white indicates the absence of links).

In this study, the quantity of network nodes is 400, thus the $2\ln\left(M\right)$ is 11.98. In Table 1, when $X_{th}$ is below 0.6, the network density varies in [3.6% 39.3%] and the mean node degree exceeds 11.98, which are most consistent with the features of the FBN in humans. In addition, when $X_{th}$ = 0.2, the SD values are smallest. Hence, the value of $X_{th}$ was taken as 0.2 in this study, and the edge links of the FBN for subject 12255 were obtained, as presented in Figure 3.

**Figure 4:**
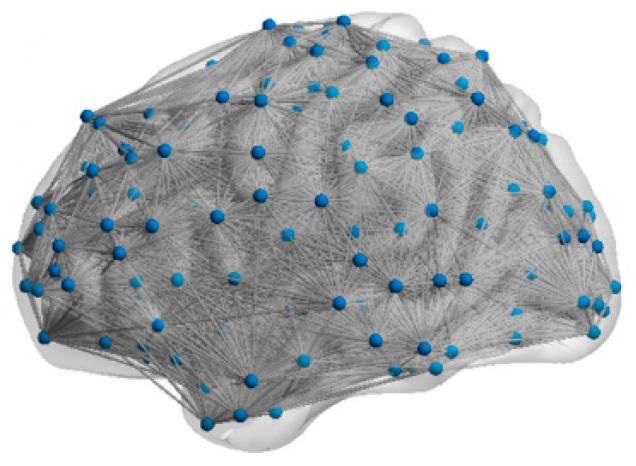
Visualization of the topology.

(3) Network topology: We quantitatively characterized the topological features of the FBNs by computing their SW feature σ and SF feature γ. A network exhibits the SW feature when σ is greater than one [43] and exhibits the SF feature when γ is between two and three [44]. In this study, the topological features of five subjects’ FBNs (mean σ = 1.83, mean γ = 2.19) demonstrated these FBNs have both SW and SF features. Based on these findings, we chose the FBN with both the SW and SF feature for our fMRI-SNN. The visualization of the topology for subject 12255 is presented in Figure 4.

2. Izhikevich neuron model

The Izhikevich neuron model was used to represent the network nodes in the fMRI-SNN. It is expressed as follows [10]:

(2) $$\left\{\begin{array}{c} dv/dt=0.04v^{2}+5v+140-u+I_{ex}+I_{g}\\ du/dt=a\left(bv-u\right)\\ if\;v\geq 30,\;\;then\left\{\begin{array}{c} v=c\\ u=u+d \end{array}\right. \end{array}\right.$$

where v denotes the MP, and u corresponds to the recovery variable related to the MP. The model incorporates two current components, as follows: $I_{ext}$, denoting the exterior input current, and $I_{g}$, denoting the synaptic current. a, b, c, and d are tunable parameters that enable the simulation of distinct firing patterns of both excitatory and inhibitory neurons.

3. Synaptic plasticity model

A synaptic plasticity model with STD was employed to denote the edges in the fMRI-SNN. This is defined as follows [45]:

(3) $$I_{syn}=g_{syn}r\left(V_{syn}-V_{post}\right)$$

where $I_{syn}$ represents the synaptic current; $V_{syn}$ represents the reversible MP; $V_{post}$ is the post-synaptic MP; $g_{syn}$ is the synaptic weight; and r reflects the change in neurotransmitter concentrations H, which is expressed as follows:

(4) $$\left\{\begin{array}{c} dr/dt=\alpha H\left(1-r\right)-\beta r\\ H={\left[1+\exp(-V_{pre}(t-t_{d}))\right]}^{-1} \end{array}\right.$$

where $V_{pre}$ is the pre-synaptic MP. The ES weight $g_{ex}$ and IS weight $g_{in}$ are regulated by the following rules [46]:

When a post-neuron j does not have an excitation potential from a pre-neuron i, $g_{ex}$ and $g_{in}$ are defined and expressed as follows:

(5) $$\left\{\begin{array}{c} \tau_{ex}(dg_{ex}/dt)=-g_{ex}\\ \tau_{in}(dg_{in}/dt)=-g_{in} \end{array}\right.$$

When a post-neuron j obtains excitation potential from a pre-neuron i, $g_{ex}$ and $g_{in}$ are defined as follows:

(6) $$\left\{\begin{array}{c} g_{ex}\left(t\right)\to g_{ex}\left(t\right)+{\bar{g}}_{ex}\\ {\bar{g}}_{ex}\to w_{∆t}*g_{max} \end{array},\;w_{∆t}=\left\{\begin{array}{c} A_{+}\exp\left(∆t/\tau_{+}\right),∆t<0\\ {-A}_{-}\exp\left(-∆t/\tau_{-}\right),∆t\geq 0 \end{array}\right.\right.$$

(7) $$\left\{\begin{array}{c} g_{in}\left(t\right)\to g_{in}\left(t\right)+{\bar{g}}_{in}\\ {\bar{g}}_{ex}\to m_{∆t}*g_{max} \end{array}\right.,m_{∆t}=\left\{\begin{array}{c} {-B}_{+}\exp\left(∆t/\tau_{+}\right),∆t<0\\ B_{-}\exp\left(-∆t/\tau_{-}\right),∆t\geq 0 \end{array}\right.$$

where ∆t is the duration between the pre-synaptic and the post-synaptic spikes.

#### 2.1.2. Construction of SNNs with Distinct Topologies

To study the influence of the topology on the damage resistance, we also constructed SWSNN and SFSNN. Both models retained an identical network size, neuron model, and synaptic plasticity model as the fMRI-SNN, with topology serving as the only variable. The topologies for SWSNN and SFSNN were obtained as described below:

1. SWSNN

**Figure 5:**
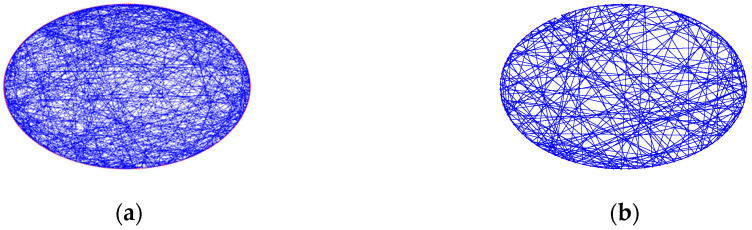
Topological visualization of the SWSNN. (**a**) 400 nodes; (**b**) 100 nodes.

The topology of the SWSNN was obtained using the WS algorithm [20] through the following steps: (1) a regular ring network consisting of M nodes is formed, in which every node connects to its K nearest neighbors; (2) edges are randomly linked according to a reconnection probability $p_{r}$. For comparison with the fMRI-SNN (σ = 1.83), we adjusted the SWSNN parameters (K = 10, $p_{r}$ = 0.6) to achieve a same SW feature (σ = 1.83), as presented in Figure 5a. To improve topological visualization, we decreased the quantity of nodes from 400 to 100 in Figure 5b.

2. SFSNN

**Figure 6:**
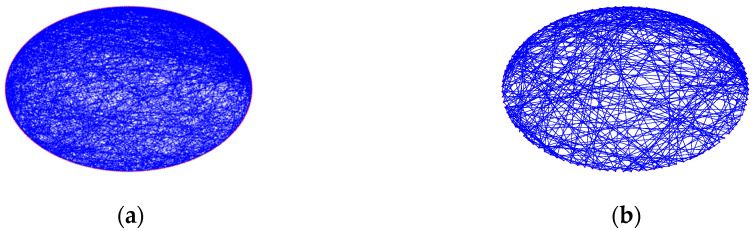
Topological visualization of the SFSNN; (**a**) 400 nodes; (**b**) 100 nodes.

The topology of the SFSNN was obtained with the BA algorithm [21], using the following steps: (1) a network with $m_{0}$ nodes that are fully linked is formed; (2) a node with $m_{e}$ edges is then inserted into the network, where $m_{e}$ is relative to the quantity of edges of the existent nodes. For comparison with the fMRI-SNN (γ = 2.19), we adjusted the SFSNN parameters ($m_{0}$ = 8, $m_{e}$ = 3) to achieve a same SF feature (γ = 2.19), as presented in Figure 6a. To improve the topological visualization, we decreased the quantity of nodes from 400 to 100 in Figure 6b.

### 2.2. Speech Recognition Framework Based on SNNs

**Figure 7:**
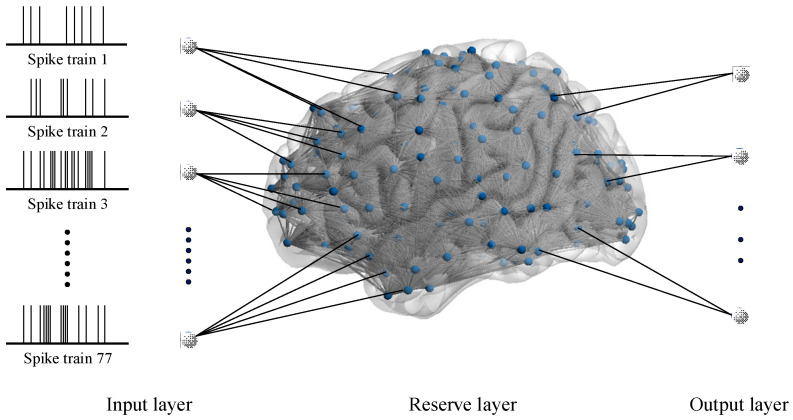
LSM framework based on an SNN.

To certify the damage resistance of fMRI-SNN, we conduct SR tasks. The LSM, which serves as an SR framework, is organized into three distinct layers, as presented in Figure 7.

From Figure 7, the input layer is populated by the Izhikevich neuron models to process preprocessed spike trains. Critically, the quantity of neurons in this layer is dynamically adapted to match the quantity of frequency bands obtained from multiscale decomposition of speech signals. The reserve layer, implemented as an SNN, constructed as described above, exhibits complex nonlinear dynamics. The output layer is populated by Izhikevich neuron models, with the population size determined by the quantity of target speech categories. The synaptic connections between these two layers are optimized through remote supervised learning (ReSuMe) by minimizing the difference between the actual firing sequence and the desired firing sequence, an efficient training algorithm specifically designed for SNN.

#### 2.2.1. Preprocessing of Speech Signal

**Figure 8:**
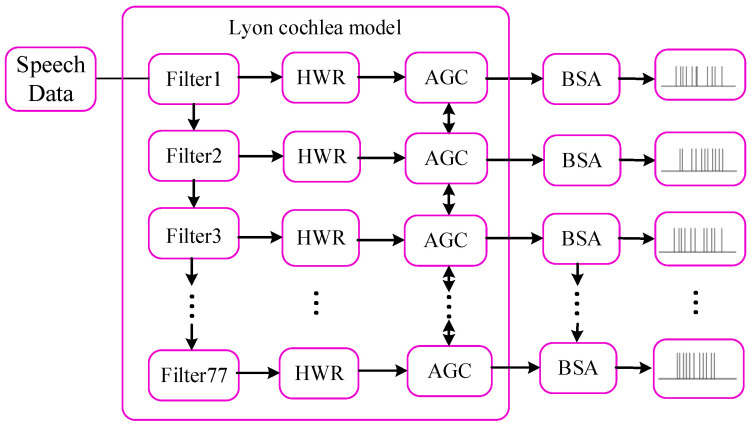
Flow chart for speech signal preprocessing.

The original speech data are in the form of an analog signal, and the neuron models in the input layer only receive the spike trains. Thus, the original speech data were preprocessed into spike trains by employing the Lyon cochlea model [47] and Ben’s spiker algorithm (BSA) [48]. Figure 8 presents a flowchart that illustrates the speech signal preprocessing.

In Figure 8, the original speech signal was first processed by the Lyon cochlea model, including filter, half wave rectifier (HWR), and automatic gain control (AGC). The signal was handled by 77 cascaded bandpass filters, each of which extracts a specific frequency band of the speech spectrum. Filter 1 captures the highest band, while Filter 77 captures the lowest band. The output of each filter is then demodulated using an HWR, which lowpasses the signal to fit within the speech range detected by the human auditory system. The AGC modules subsequently normalize the signal amplitudes by dynamically adjusting their energy levels to fall within the optimal perceptual range of human auditory sensitivity. Finally, BSA was used to encode these signals with distinct levels of energy into spike trains with distinct instantaneous firing rates.

#### 2.2.2. Firing Patterns of SNNs Triggered by Speech Signal

**Figure 9:**
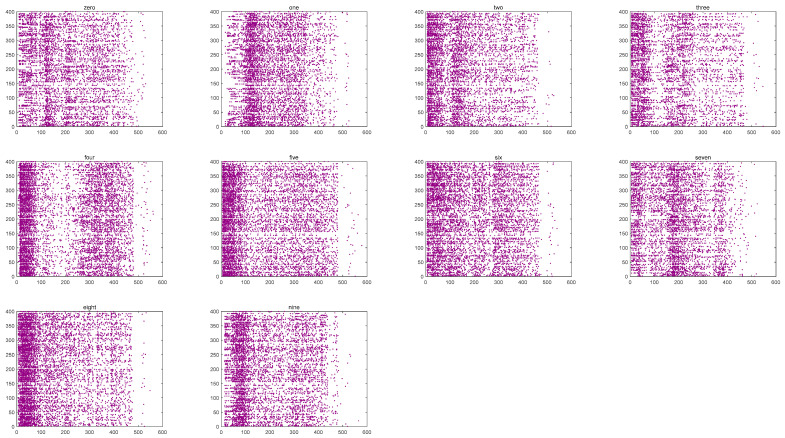
Firing patterns of the fMRI-SNN.

Once the input layer receives the spike trains, they are passed to the reserve layer as input currents, causing changes in neural activity. Figure 9 illustrates the firing patterns of the fMRI-SNN over 600 ms, triggered by distinct speech signals (“zero” to “nine”).

Figure 9 shows that significant distinctions in the firing patterns of the network are caused by distinct digital speech signals. The test level for the distinctions is represented by P, which is calculated by a one-way analysis of variance, focusing on the quantity of firing neurons at each time point. In this study, the result of P is below 0.01, indicating extreme distinctions of the firing patterns. We therefore employ these firing patterns as characteristics for speech recognition.

#### 2.2.3. Optimization of Synaptic Weights During Training

In the training process of SNN, dynamic optimization of synaptic weights is the core link to achieve efficient information processing and pattern recognition. A ReSuMe is employed to optimize the synaptic weights $g\left(t\right)$ among the reserve layer and output layer during training. It is expressed as follows [17]:

(8) $$dg\left(t\right)/dt=\left[s\left(t_{d}\right)-s\left(t_{a}\right)\right]\left[e_{H}+\int_{0}^{\infty }w\left(∆t\right)s\left(t_{in}-∆t\right)dt\right]$$

where $s\left(t_{a}\right)$ represents the actual firing sequence produced by the output layer during each training epoch; $s\left(t_{d}\right)$ represents the desired firing sequence based on the distinction in the firing patterns caused by distinct speech signals; $s\left(t_{in}\right)$ represents the input firing sequence; and $e_{H}$ represents the error gain coefficient (set to 0.25), and is used to balance the learning rate and stability of synaptic plasticity.

**Figure 10:**
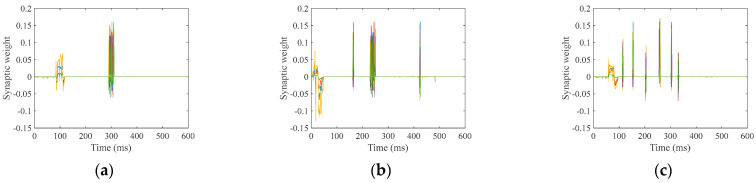
Training synaptic weights; (**a**) “zero”; (**b**) “five”; (**c**) “nine”.

The LSM processes individual utterances of the ten digits, with each full pass through the training dataset defined as one epoch for synaptic weight optimization. The ideal synaptic weight between the reserve and output layers is achieved through iterative training, and the final weights are determined after all specified training epochs are completed for each digit. Figure 10 illustrates the synaptic weights post-training, exemplified by the following speech digits: “zero”, “five”, and “nine”.

Figure 10 shows that the synaptic weights values oscillate above and below zero at the expected firing moments.

#### 2.2.4. Decision-Making Process

**Figure 11:**
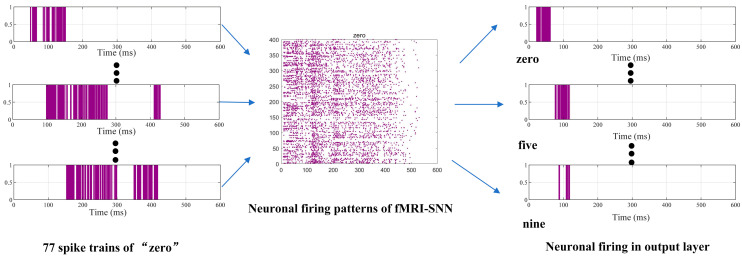
Neuronal firing activities of “zero” during decision process.

When preprocessed speech signals enter our SR framework, the neuron models of SNN generate distinct firing patterns during the decision process. The output layer’s spiking activity, modulated by training synaptic weights (reserve to output layer), determines recognition outcomes. Finally, the output neuron with the highest firing frequency is selected through a winner-takes-all mechanism, assigning its class label as the recognition result. To illustrate this process, Figure 11 presents neuronal firing activities of “zero” during the decision process.

#### 2.2.5. Time Complexity

The computational cost of our speech recognition method is evaluated through time complexity analysis. This complexity comprises firing patterns of SNNs triggered by speech signal, training synaptic weight (reserve to output layer), and output layer neuron decision.

(1) The firing patterns of SNNs triggered by speech signal

In this process, the speech spike trains undergo current-driven transmission. The time complexity for input current-induced neuronal firing is O(p), where p denotes the speech spike train count. With each neuron model connecting to, at most, n others, the time complexity for synaptic current-induced firing is O(n). The time of each speech data is t, and the total number of speech data is k. Therefore, the time complexity of neuronal firing of fMRI-SNN with n neuron models caused by speech data is O(k×t×n×(p+n)).

(2) The training synaptic weight

Each neuron n model can link up to n others, resulting in a time complexity of O(n×n) for the actual output sequence relayed by up to n other neuron models through synaptic plasticity. In contrast, the desired output sequence has a time complexity of O(1). The input sequence is processed over a duration of time t within the ReSuMe framework, with its time complexity being O(t). When utilizing ReSuMe for training synaptic weights, the time complexity becomes O((1+n×n)×t), which simplifies to O(n×n×t). Each speech data instance has a duration of t, and the count of training sets for the speech data is m. Consequently, the overall time complexity for training synaptic weights across the speech data training sets is O(m×t×n×n×t).

(3) The output layer neuron decision.

The fMRI-SNNs have n synaptic connections to the output layer, resulting in a time complexity of O(n) for computing neuronal firing in this layer. With each speech data point having a duration of t and the test sets consisting of c speech data points, the time complexity for the neurons’ decision-making process in the output layer, when evaluated against the test sets, becomes O(c×t×n).

#### 2.2.6. Scalability

To evaluate the scalability of fMRI-SNN, we assess the running time required for training the synaptic weights across various network sizes (90, 400, 980, 1024) of fMRI-SNN. The simulations were performed on a computer equipped with a 2.50 GHz CPU and 8 GB of RAM. The running times for training the synaptic weights in these fMRI-SNNs are presented in Table 2.

As shown in Table 2, the time required for training synaptic weights rises with the growth in network size. Although the network increases by a certain multiple, the increase in running time is less than that multiple, suggesting that our method demonstrates scalability for larger networks.

## 3. Results

This section starts with an introduction to the experimental configurations and the dataset applied in our experiments. Subsequently, an SR task is employed to certify the damage resistance of fMRI-SNN against stochastic attack by analyzing the SR accuracy.

### 3.1. Experimental Configurations and Dataset

The experimental configurations are introduced, including the parameters of the Izhikevich model and the synaptic plasticity model. The speech dataset is then summarized.

#### 3.1.1. Experimental Configurations

Following [49], the SNNs incorporate the Izhikevich models with a randomized 4:1 distribution ratio between excitatory and inhibitory neurons. The corresponding parameter descriptions and their values are detailed in Table 3.

Following [45,46], descriptions and values for the parameters of synaptic plasticity model are presented in Table 4.

#### 3.1.2. Speech Dataset

The TI-46 speech dataset is a benchmark in SR tasks, commonly used to certify SNN performance. For our SR experiments, we utilize the TI-46 corpus subset from the Linguistic Data Consortium (https://catalog.ldc.upenn.edu/LDC93S9, accessed on 23 June 2025), which consists of 4000 utterances from 16 speakers, each speaking 10 digits (“zero” to “nine”) 25 times. To ensure that our method was generalizable, we use 2400 utterances (16 speakers, 10 digits, 15 times) as the training set and the remaining as the test set. For each digit, the trained synaptic weights are obtained after 240 epochs, as presented in Figure 10.

### 3.2. Certification of the Damage Resistance of the fMRI-SNN

We assume the neurons are physically damaged when they suffer certain attack. For example, electromagnetic interference can cause chip damage, resulting in distorting signals or loss of information transmission in the chip [50]. When transistors that simulate the functions of neurons and synapses fail under electromagnetic interference, their neural information processing and synaptic connectivity functions are damaged, making it impossible to correctly perform signal amplification, integration, or transmission [51]. These effects are comparable to stochastically removing neurons, which disrupts information transmission and the network’s function. Thus, to simulate such damage caused by stochastic attacks, we stochastically eliminated a subset of neuron models from the SNN, along with their associated synaptic plasticity models. In this paper, the fMRI-SNN comprises 400 Izhikevich neuron models. The damage proportion $p_{d}$ ranges from 5% to 50%, with a step size of 5%, corresponding to the elimination of 20, 40, 60, 80, 100, 120, 140, 160, 180, and 200 neuron models, respectively. In this subsection, we conducted SR tasks to certify the damage resistance of our fMRI-SNN against stochastic attack by analyzing the SR accuracy. Firstly, we investigated the impact of $X_{th}$ on the damage resistance of the fMRI-SNN; secondly, we compared the damage resistance of the SNNs to distinct topologies; and finally, we verified the generalization ability of fMRI-SNN.

#### 3.2.1. The Damage Resistance of the fMRI-SNN with Distinct $X_{th}$

**Figure 12:**
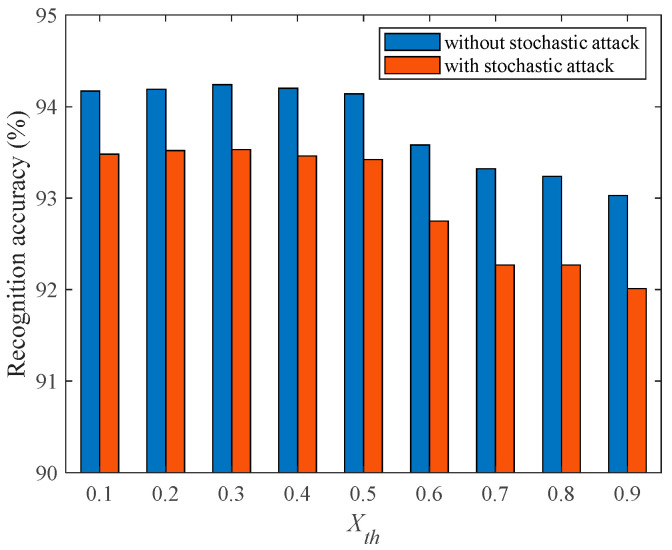
Recognition accuracy of fMRI-SNN with distinct $X_{th}$.

To investigate the impact of $X_{th}$ on the damage resistance of the fMRI-SNN, we compared the SR accuracies of fMRI-SNN with and without stochastic attack for $X_{th}$ in the range of 0–0.9 with a step size of 0.1. Taking $p_{d}$ = 25% as an example, the SR accuracy of fMRI-SNN with distinct $X_{th}$ is presented in Figure 12.

Figure 12 shows that for all $X_{th}s,$ the SR accuracy of fMRI-SNN with stochastic attack decreases compared to without stochastic attack, and when $X_{th}$ is 0.2, the SR accuracies of fMRI-SNN with stochastic attack decreases the least, indicating that the damage resistance of the fMRI-SNN with $X_{th}$ of 0.2 is the best. These results related to the consistency between the mean node degree and network density of the constructed fMRI SNN and the bio-FBN when $X_{th}$ is 0.2, verifying that a brain-like model with bio-plausibility can enhance its damage resistance. The optimal $X_{th}$ determined based on damage resistance is the same as the optimal $X_{th}$ determined based on topological features and the SD of bio-FBN in Section 2.1.1; furthermore, both the of $X_{th}$s are 0.2, verifying that a brain-like model with bio-plausibility can enhance its damage resistance.

#### 3.2.2. The Comparison of the Damage Resistance of the SNNs with Distinct Topologies

**Figure 13:**
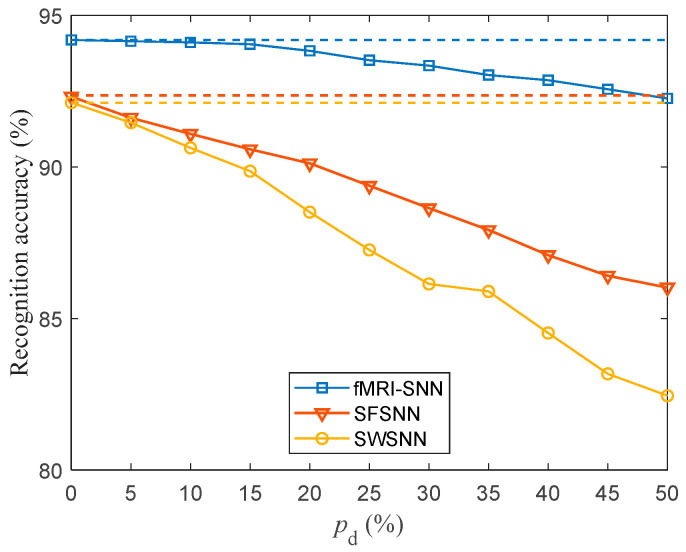
Recognition accuracy for SNNs with distinct topologies against stochastic attack (dashed lines denote the recognition accuracy of SNNs without stochastic attack).

To investigate the impact of topologies on the damage resistance of the SNNs, we compared the SR accuracies of SNNs with distinct topologies. For $p_{d}$ in the range of 5–50% with a step size of 5%, the SR accuracy of SNNs with distinct topologies against stochastic attack is presented in Figure 13.

Figure 13 shows the following: (1) the SR accuracies of SNNs with distinct topologies decrease as $p_{d}$ increases; (2) our fMRI-SNN demonstrates a marked superiority in SR accuracy over both SWSNN and SFSNN against stochastic attack.

The discrepancy in the SR accuracy of SNN with and without stochastic attack serves as an indicator of its damage resistance. The smaller the discrepancy in SR accuracy, the superior the damage resistance of SNN. The discrepancy in the SR accuracy of SNNs with distinct topologies with and without stochastic attack is presented in Table 5.

Table 5 shows that (1) the discrepancy in the SR accuracy of distinct SNNs increases as $p_{d}$ increases; (2) the mean discrepancy in the SR accuracy of distinct SNNs for $p_{d}$ in the range [5%, 50%] is as follows: fMRI-SNN is 0.82%, SWSNN is 5.13%, and SFSNN is 3.47%, indicating that the damage resistance of the fMRI-SNN is the best; and (3) the mean discrepancy in SR accuracy of fMRI-SNN for $p_{d}$ in the range [5%, 30%] is 0.36%, indicating that when $p_{d}$ is below 30%, the damage resistance of fMRI-SNN is almost the same as that without stochastic attack.

#### 3.2.3. The Generalization Ability of fMRI-SNN

To verify the generalization ability of the fMRI-SNN in SR tasks and avoid potential bias of the TI-46 dataset on the results, we conducted the same experimental procedure using the Free Spoken Digit Dataset (https://github.com/Jakobovski/free-spoken-digit-dataset, accessed on 23 June 2025). The Free Spoken Digit Dataset consists of 2000 utterances from 4 speakers, each speaking 10 digits (“zero” to “nine”) 50 times. To ensure that our method was generalizable, we use 1200 utterances (4 speakers, 10 digits, 30 times) as the training set and the remaining as the test set.

**Figure 14:**
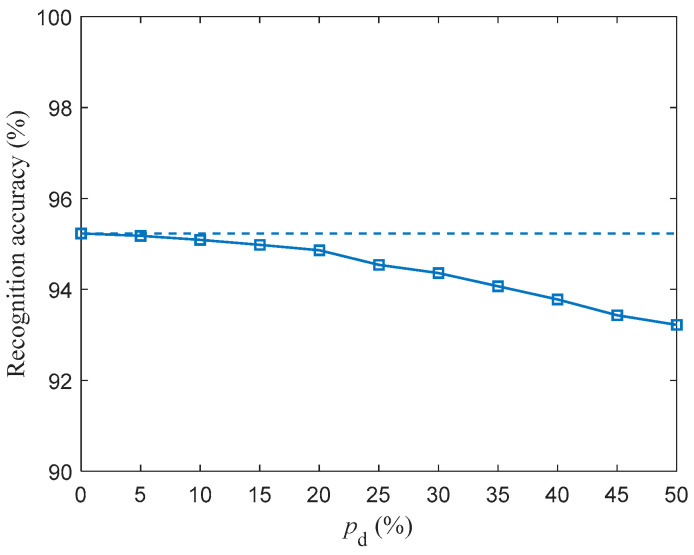
Recognition accuracy of fMRI-SNN against stochastic attack (dashed lines denote the recognition accuracy of fMRI-SNN without stochastic attack).

For $p_{d}$ in the range of 5–50% with a step size of 5%, the SR accuracy of fMRI-SNN against stochastic attack is investigated, and the corresponding result is presented in Figure 14.

Figure 14 shows that the SR accuracies of fMRI decrease as $p_{d}$ increases. The discrepancy in the SR accuracy of fMRI-SNN with and without stochastic attack is presented in Table 6.

Table 6 shows that (1) the discrepancy in the SR accuracy of fMRI-SNN increases as $p_{d}$ increases; (2) the mean discrepancy in the SR accuracy of fMRI-SNN for $p_{d}$ in the range [5%, 30%] is 0.4%, indicating that when $p_{d}$ is below 30%, the damage resistance of fMRI-SNN is almost the same as that without stochastic attack. These results, based on the Free Spoken Digit Dataset, are consistent with those based on the TI-46 dataset, verifying that our fMRI SNN has the ability to be generalized.

To summarize, these results suggest that (1) the fMRI-SNN we constructed has better bio-plausibility; (2) the damage resistance of our fMRI-SNN exceeds that of SWSNN and SFSNN, especially when $p_{d}$ is below 30%, the damage resistance of fMRI-SNN is almost the same as that without stochastic attack, meaning that our method improves the damage resistance of a brain-like model; and (3) the damage resistance of fMRI-SNN is verified in both the TI-46 dataset and the Free Spoken Digit dataset, meaning that our fMRI-SNN has the ability to be generalized.

## 4. Discussion

To reveal the damage-resistant mechanism of our fMRI-SNN, we analyzed its neural electrical features, its adaptive modulation of synaptic plasticity, and its topological features against stochastic attack.

### 4.1. Neural Electrical Features

We revealed the damage-resistant mechanism of the fMRI-SNN from the perspective of neural electrical features, including the relative change in the firing rate and the correlation between the MPs, which represent the change in neural electrical activity against stochastic attack.

#### 4.1.1. Relative Change Rate in Firing Rate

**Figure 15:**
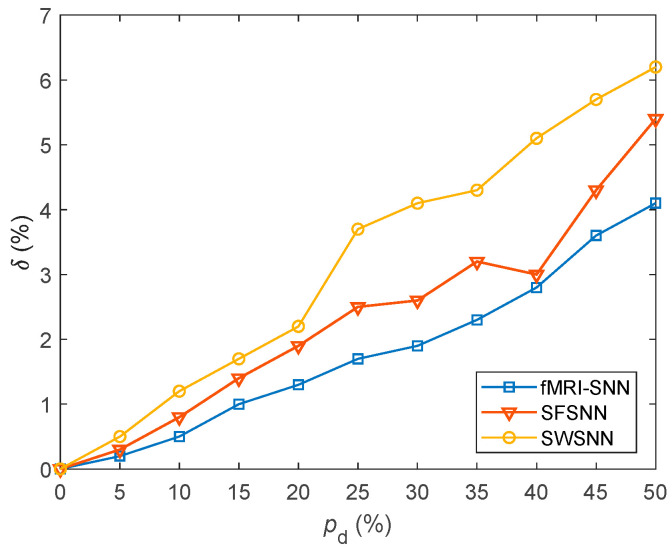
Results for δ for SNNs with distinct topologies against stochastic attack.

The $\delta_{i}$ represents the relative change rate of a neuron model’s firing rate, reflecting the discrepancy of firing rate with and without stochastic attack. δ represent the mean of $\delta_{i}$ for all neurons, which is defined as follows:

(9) $$\delta =\frac{1}{N}\sum_{i=1}^{N}\delta_{i}=\frac{1}{N}\sum_{i=1}^{N}\frac{\left|f_{iB}-f_{iA}\right|}{f_{iA}}*100\%$$

where $f_{iA}$ and $f_{iB}$ represent the firing rates of a neuron with and without stochastic attack, respectively. A lower value of δ signifies a lesser change in neural electrical activity. For $p_{d}$ in the range of 5–45% with a step size of 5%, the value of δ for SNNs with fMRI-SNN, SWSNN, and SFSNN against stochastic attack are compared in Figure 15.

Figure 15 shows that compared to both SWSNN and SFSNN, the fMRI-SNN exhibits significantly lower δ values as $p_{d}$ increases, indicating that our fMRI-SNN has the most stable neural electrical activity against stochastic attack.

#### 4.1.2. Correlation Between Membrane Potentials

**Figure 16:**
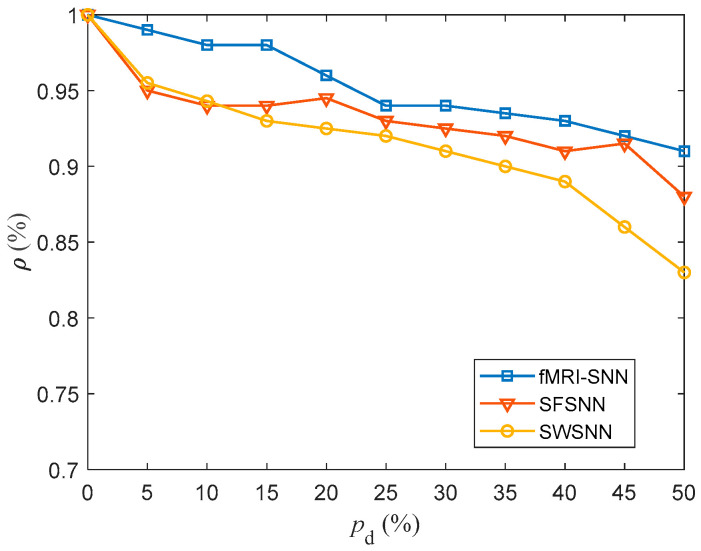
Results for ρ for SNNs with distinct topologies against stochastic attack.

The $\rho_{i}$ represents the correlation between MPs of a neuron with and without stochastic attack. ρ represents the mean $\rho_{i}$ for all neurons, which is expressed as follows:

(10) $$\rho =\frac{1}{N}\sum_{i=1}^{N}\rho_{i}=\frac{1}{N}\sum_{i=1}^{N}\frac{\sum_{t=t_{1}}^{t_{2}}x_{iA}\left(t\right)x_{iB}\left(t\right)}{\sqrt{\sum_{t=t_{1}}^{t_{2}}x_{iA}^{2}\left(t\right)\sum_{t=t_{1}}^{t_{2}}x_{iB}^{2}\left(t\right)}}$$

where $x_{iA}$ and $x_{iB}$ represent the MPs of a neuron model with and without stochastic attack, respectively. $\left[t_{1},\;{\;t}_{2}\right]$ is the duration of the simulation. The higher the ρ, the less change there is in neural electrical activity. In this paper, the $\left[t_{1},\;{\;t}_{2}\right]$ was taken as 1000 ms. For $p_{d}$ in the range of 5–45% with a step size of 5%, the value of ρ for SNNs against stochastic attack are compared in Figure 16.

Figure 16 shows that compared to both SWSNN and SFSNN, the fMRI-SNN exhibits significantly higher ρ values as $p_{d}$ increases, indicating that our fMRI-SNN has the most stable neural electrical activity against stochastic attack.

#### 4.1.3. Correlation Analysis

To further discuss the relationship between the SR accuracies and the change in neural electrical activity of SNNs against stochastic attack, a Pearson correlation analysis was performed. This analysis examined the correlation coefficient (R) between SR accuracy (A) and neural electrical features (B) of the SNN against stochastic attacks, as defined in Equation (1). According to R, the *t*-test was employed to mark the significance level of correlation, which is described as follows:

(11) $$t_{test}=R/\sqrt{1-R^{2}/\left(n-2\right)}$$

A 0.01 level of significance is denoted as “**”, and a 0.05 level is denoted as “*”.

The correlations between the SR accuracy and the neural electrical features (δ and ρ) of fMRI-SNN, SWSNN, and SFSNN against stochastic attack are given in Table 7.

The results in Table 7 show a significant correlation between SR accuracies and neural electrical features (each $t_{test}$ < 0.01). This means that the change in neural electrical activity is the interior manifestation of brain-like models, and it corresponds to the damage resistance of brain-like models for the SR task.

### 4.2. Adaptive Modulation of Synaptic Plasticity

To address the reason for the lesser change in the neural electrical activity of the fMRI-SNN against stochastic attack, we revealed the damage-resistant mechanism from the adaptive modulation of synaptic plasticity perspective.

#### 4.2.1. Impact of Synaptic Plasticity on the Damage Resistance

**Figure 17:**
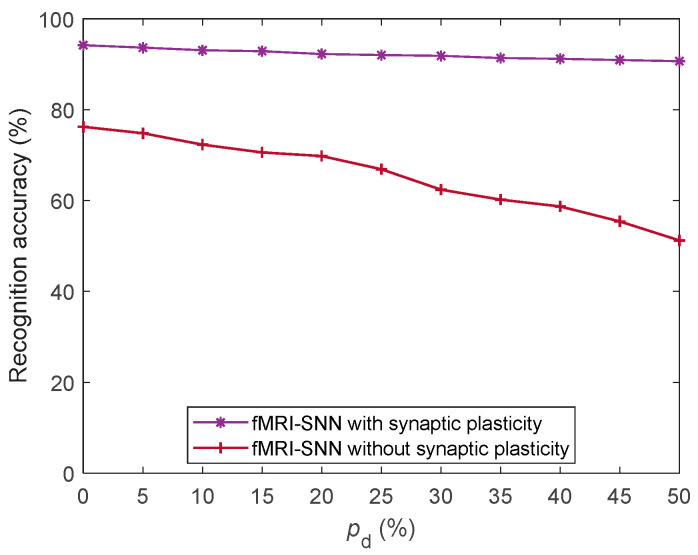
SR accuracy of fMRI-SNN with and without synaptic plasticity against stochastic attack.

To assess the impact of synaptic plasticity on the damage resistance of the fMRI-SNN, we compared its performance with and without synaptic plasticity against stochastic attack, as presented in Figure 17.

Figure 17 shows that the fMRI-SNN with synaptic plasticity significantly exceeds that without synaptic plasticity in terms of the SR accuracy against stochastic attack, implying that the damage resistance of the SNN relies critically on the adaptive modulation of synaptic plasticity.

#### 4.2.2. Relationship Between Synaptic Plasticity and Damage Resistance

To reveal the damage-resistant mechanism of the fMRI-SNN from the adaptive modulation of synaptic plasticity perspective, we presented the dynamics of both the mean synaptic weight (MSW) and the neural electrical features of the fMRI-SNN against stochastic attack, and investigated the correlation between them.

1. Dynamics of the mean synaptic weight

**Figure 18:**
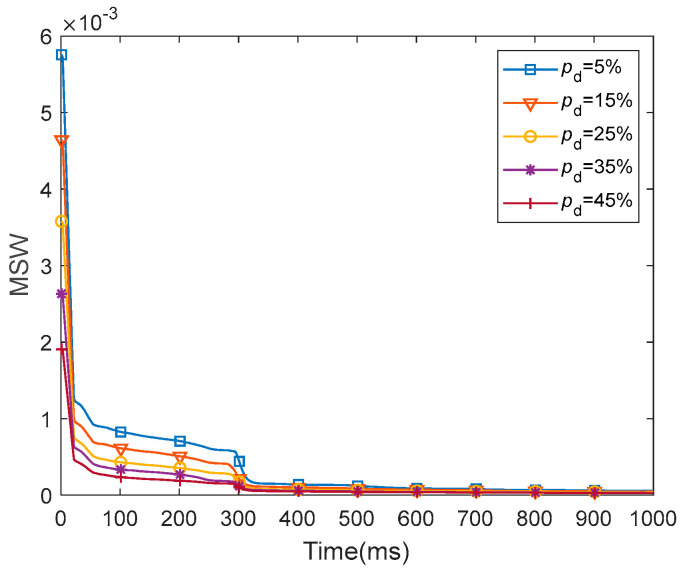
Dynamics of the MSW for fMRI-SNN against stochastic attack.

The MSW represents the mean weight for all of the synapses in the entire network. We presented the dynamics of the MSW of the fMRI-SNN against stochastic attack within 1000 ms, as presented in Figure 18.

Figure 18 shows that for distinct values of $p_{d}$, the dynamics of MSW for fMRI-SNN against stochastic attack present a similar trend, which is as follows: the MSW exhibits an obvious decline during the initial 300 ms, subsequently stabilizing between 300 ms and 1000 ms. This result reflects the self-adaptive modulation of synaptic plasticity process of synaptic plasticity in response to stochastic attacks.

2. Dynamics of the neural electrical features

**Figure 19:**
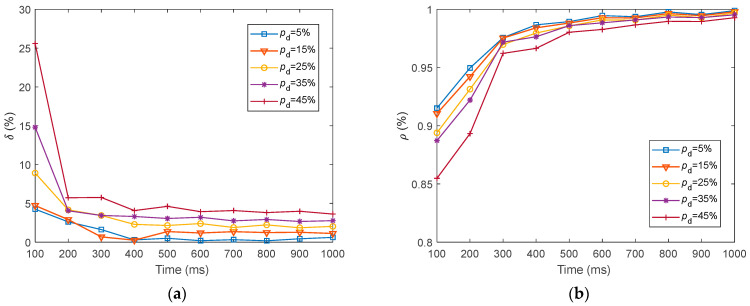
Dynamics of the neural electrical features for fMRI-SNN against stochastic attack. (a) δ; (b) ρ.

We presented the dynamics of the neural electrical features (δ and ρ) for fMRI-SNN against stochastic attack within 1000 ms, as presented in Figure 19.

Figure 19 shows that for distinct values of $p_{d}$, the dynamics of δ and ρ for fMRI-SNN against stochastic attack present a similar trend, which is as follows: the δ and ρ exhibit an obvious decline during the initial 300 ms, subsequently stabilizing between 300 ms and 1000 ms.

3. Correlation analysis

We carried out a Pearson correlation analysis defined in Equation (1) to explore the relationship between the dynamics of MSW and the neural electrical features (δ and ρ) against stochastic attack within 1000 ms. A and B in Equation (1) are the MSW and the neural electrical features of the SNN against stochastic attack, respectively. The results are presented in Table 8.

From Table 8, the MSWs are significantly correlated with δ and ρ at the 0.01 level.

To summarize, by comparing the SR accuracy of fMRI-SNNs with and without synaptic plasticity, along with the correlation between the MSW and δ and ρ, we conclude that the inherent determinant impacting the damage resistance is the synaptic plasticity.

### 4.3. Topological Features

To address the reason for the impact of the topology on the damage resistance of SNN, we revealed the damage-resistant mechanism from the topological perspective, including the mean weighted clustering coefficient (MWCC) and the mean weighted shortest path length (MWSPL).

#### 4.3.1. Mean Weighted Clustering Coefficient

The MWCC ($\tilde{C}$) is a key topological indicator for measuring the local efficiency of information transfer in a network, indicating the degree of compactness among nodes within a network. The larger the value of $\tilde{C}$, the higher the local efficiency. This can be expressed as follows [52]:

(12) $$\tilde{C}=\frac{1}{N}\sum_{i=1}^{N}\left(\frac{1}{s_{i}\left(k_{i}-1\right)}\sum_{j,k}\frac{\left(g_{ij}+g_{ik}\right)}{2}a_{ij}a_{jk}a_{ki}\right)$$

where $g_{ij}$ and $g_{ik}$ represent the synaptic weights; $k_{i}$ represents the of node degree; $s_{i}$ represents the node strength; and $a_{ij}$, $a_{jk}$, and $a_{ki}$ represent the adjacency matrix.

**Figure 20:**
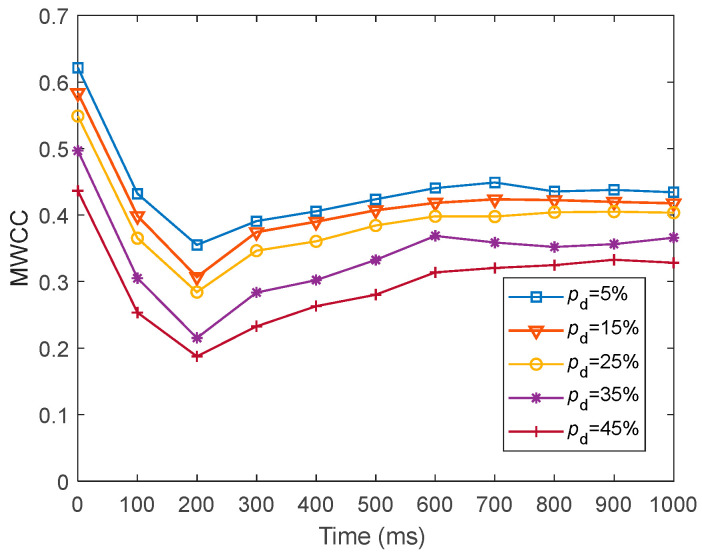
Dynamics of $\tilde{C}$ for fMRI-SNN against stochastic attack with distinct values of $p_{d}$.

To present the local efficiency of information transfer in fMRI-SNN against stochastic attack, we presented the dynamics of $\tilde{C}$ for fMRI-SNN against stochastic attack, with distinct values of $p_{d}$ within 1000 ms, as presented in Figure 20.

Figure 20 shows that for distinct values of $p_{d}$, the dynamics of $\tilde{C}$ for fMRI-SNN against stochastic attack present an overall decreasing trend, suggesting that the local efficiency of information transfer in the fMRI-SNN decreases as $p_{d}$ increases.

**Figure 21:**
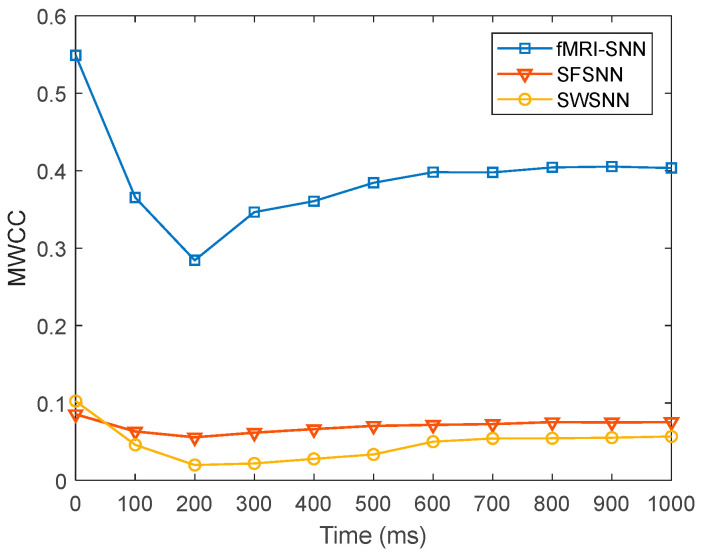
Dynamics of $\tilde{C}$ for SNNs against stochastic attack.

To further present the local efficiency of information transfer in SNNs with distinct topologies, we compared the dynamics of $\tilde{C}$ for SNNs with distinct topologies at $p_{d}$ = 25%, as presented in Figure 21.

Figure 21 shows that when $p_{d}$ is 25%, the fMRI-SNN exhibits significantly higher$\;\tilde{C}$ values compared to both SWSNN and SFSNN, implying superior local efficiency of information transfer efficiency for our fMRI-SNN.

#### 4.3.2. Mean Weighted Shortest Path Length

The MWSPL ($\tilde{L}$) is a key topological indicator for measuring the global efficiency of information transfer in a network, indicating the shortest distance between node pairs. The smaller the value of $\tilde{L}$, the higher the global efficiency. This can be expressed as follows [53]:

(13) $$\tilde{L}=\frac{1}{N\left(N-1\right)}\begin{array}{c} min\\ \Upsilon \left(i,j\right)\in \Gamma \left(i,j\right) \end{array}\left[\sum_{m,n\in \Upsilon \left(i,j\right)}\frac{1}{g_{mn}}\right]$$

where $g_{mn}$ represents the weight of synapses in fMRI-SNN; $\Upsilon \left(i,j\right)$ represents the shortest path between node pairs; and $\Gamma \left(i,j\right)$ represents the possible paths between node pairs.

**Figure 22:**
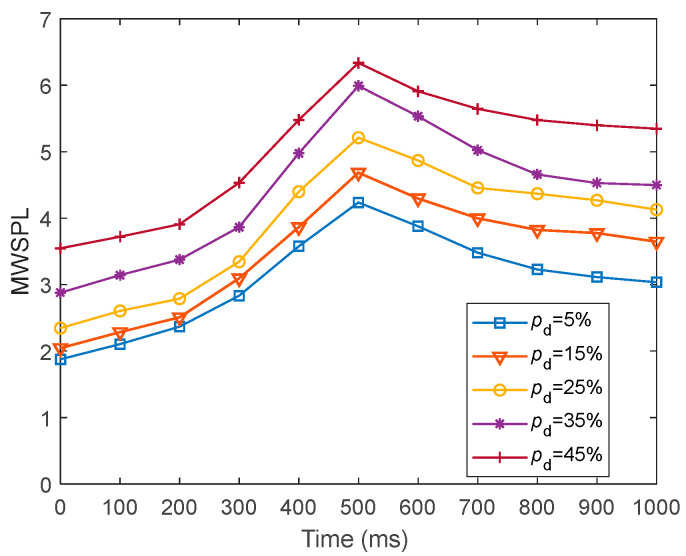
Dynamics of $\tilde{L}$ for fMRI-SNN against stochastic attack with distinct values of $p_{d}$.

To present the global efficiency of information transfer in fMRI-SNN against stochastic attack, we presented the dynamics of $\tilde{L}$ for fMRI-SNN against stochastic attack with distinct values of $p_{d}$ within 1000 ms, as presented in Figure 22.

Figure 22 shows that for distinct values of $p_{d}$, the dynamics of $\tilde{L}$ for fMRI-SNN against stochastic attack present an overall increasing trend, suggesting that the global efficiency of information transfer in the fMRI-SNN decreases as $p_{d}$ increases.

**Figure 23:**
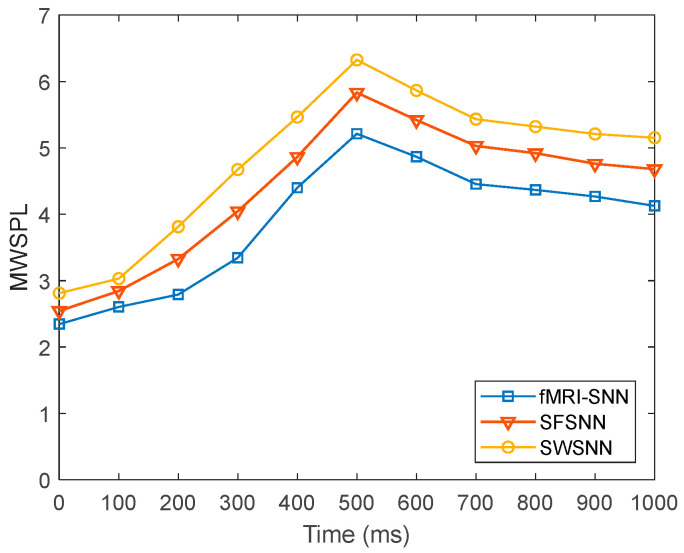
Dynamics of $\tilde{L}$ for SNNs against stochastic attack.

To further present the global efficiency of information transfer in SNNs with distinct topologies, we compared the dynamics of $\tilde{L}$ for SNNs with distinct topologies at $p_{d}$ = 25%, as presented in Figure 23.

Figure 23 shows that when $p_{d}$ is 25%, the fMRI-SNN exhibits significantly lower $\tilde{L}$ values compared to both SWSNN and SFSNN, implying superior global transfer of information efficiency for our fMRI-SNN.

To summarize, our analysis of the topological features, as indicated by $\tilde{C}$ and $\tilde{L}$, reveals that the fMRI-SNN exhibits a superior performance compared to both SFSNN and SWSNN, supporting our findings for the damage resistance. This suggests that the topological features are a determinant impacting the damage resistance of an SNN.

## 5. Conclusions

We built an fMRI-SNN with the topology constrained by FBN from human fMRI data, with the aim of improving the bio-plausibility of brain-like models. We employed SR accuracy to certify the damage resistance of our fMRI-SNN against stochastic attack. The results indicated that the damage resistance of the fMRI-SNN exceeded that of SWSNN and SFSNN. Especially, the damage resistance of the fMRI-SNN against stochastic attack is almost the same as that without stochastic attack when the $p_{d}$ is below 30%. These findings mean that our method improves the damage resistance of brain-like models. We further discussed the neural electrical features, the adaptive modulation of synaptic plasticity, and the topological features of the fMRI-SNN against stochastic attack to reveal the damage-resistant mechanism. It was implied that the change in neural electrical activity is interior manifestation corresponding to the damage resistance of SNNs for SR tasks, that synaptic plasticity is the inherent determinant of damage resistance, and that the topology was a determinant impacting the damage resistance.

Our results indicate that the brain-like model with bio-plausibility can enhance its damage resistance. In future work, we will implement our proposed bio-plausible brain-like model in hardware platforms such as FPGA, and verify its damage resistance on diverse datasets related to pattern recognition in real electromagnetic environments. In addition, FBNs exhibit topological changes such as significantly shortened feature path lengths and increased clustering coefficients during cognitive task execution [54,55], indicating that the topological characteristics of FBN based on neuroimaging data during cognitive tasks become better. Thus, we will construct a brain-like network based on task-specific fMRI data during auditory processing tasks, and investigate its SR performance.

## Figures and Tables

**Table 1:** Mean and SD of network density and mean node degree.

$X_{th}$	Network Density	Mean Node Degree
0.1	0.3102 ± 0.0138	130.7561 ± 5.7235
0.2	0.2236 ± 0.0063	82.3174 ± 3.0978
0.3	0.1983 ± 0.0199	41.6176 ± 5.5473
0.4	0.0752 ± 0.0102	22.8206 ± 3.8430
0.5	0.0321 ± 0.0072	11.2529 ± 2.0578
0.6	0.0183 ± 0.0029	5.0960 ± 1.3839
0.7	0.0061 ± 0.0018	2.2584 ± 0.6438
0.8	0.0019 ± 0.0005	0.4673 ± 0.1865
0.9	0.0011 ± 0.0001	0.0398 ± 0.0098

**Table 2:** Running time of the training synaptic weights.

Network size	90	400	980	1024
Running time (s)	124.81	287.26	370.14	447.32

**Table 3:** Parameters of neuron models.

Parameter	Instruction	Value
a	Temporal scale of u	Excitement: 0.02
		Inhibition: 0.02
b	Sensitivity of u to sub-threshold fluctuations of v	Excitement: 0.20
		Inhibition: 0.25
c	Reinitialize value of v	Excitement: −65
		Inhibition: −65
d	Reinitialize value of v	Excitement: 8
		Inhibition: 2

**Table 4:** Parameters of the synaptic plasticity model.

Parameter	Description	Value
E	Reversible synaptic potential	Excitement: 0 mV
		Inhibition: −70 mV
α	Forward rate constant of neurotransmitter	Excitement: 2
		Inhibition: 0.9
β	Reverse rate constant of neurotransmitter	Excitement: 1
		Inhibition: 0.1
$g_{max}$	Maximum value of the synaptic weight	0.015
$\tau_{ex}$	Decay constant of the ES weight	3 ms
$\tau_{in}$	Decay constant of the IS weight	5 ms
$A_{+}$	Greatest modification amount with increased the ES weight	0.1
$A_{-}$	Least modification amount with decreased ES weight	0.105
$B_{+}$	Greatest modification amount with increased the IS weight	0.02
$B_{-}$	Least modification amount with decreased IS weight	0.03
$\tau_{+}$	Intervals between pre-synaptic and post-synaptic firing with increased synaptic weights	20 ms
$\tau_{-}$	Intervals between pre-synaptic and post-synaptic firing with decreased synaptic weights	20 ms

**Table 5:** Discrepancy in SR accuracy of SNNs with distinct topologies without and with stochastic attack.

$p_{d}\;$(%)	5	10	15	20	25	30	35	40	45	50
fMRI-SNN	0.04%	0.08%	0.14%	0.36%	0.67%	0.85%	1.16%	1.33%	1.63%	1.93%
SWSNN	0.66%	1.49%	2.26%	3.61%	4.86%	5.98%	6.23%	7.60%	8.95%	9.67%
SFSNN	0.74%	1.27%	1.78%	2.24%	2.98%	3.72%	4.44%	5.27%	5.95%	6.34%

**Table 6:** Discrepancy in SR accuracy of fMRI-SNN without and with stochastic attack.

$p_{d}\;$(%)	5	10	15	20	25	30	35	40	45	50
Discrepancy	0.05%	0.24%	0.25%	0.37%	0.69%	0.87%	1.16%	1.45%	1.8%	2.01%

**Table 7:** Correlations between SR accuracy and neural electrical features.

Network	Neural Electrical Features	
	δ	ρ
fMRI-SNN	−0.953 **	0.971 **
SWSNN	−0.932 **	0.955 **
SFSNN	−0.948 **	0.967 **

Note: a 0.01 level of significance is denoted as “**”.

**Table 8:** Correlations between the dynamics of MSW and neural electrical features.

$p_{d}$ (%)	5	15	25	35	45
δ	0.943 **	0.925 **	0.937 **	0.950 **	0.952 **
ρ	−0.930 **	−0.959 **	−0.946 **	−0.967 **	−0.972 **

Note: a 0.01 level of significance is denoted as “**”.

## Data Availability

The original contributions presented in this study are included in the article. Further inquiries can be directed to the corresponding author(s).

## References

[1] Wu X., Dang B., Zhang T., Wu X., Yang Y. (2024). Spatiotemporal audio feature extraction with dynamic memristor-based time-surface neurons. Sci. Adv..

[2] Han J., Li Z., Zheng W., Zhang Y. (2020). Hardware implementation of spiking neural networks on FPGA. Tsinghua Sci. Technol..

[3] Liu X., Wang F., Su J., Zhou Y., Ramakrishna S. (2022). Bio-inspired 3D artificial neuromorphic circuits. Adv. Funct. Mater..

[4] Hong Q., Chen H., Sun J., Wang C. (2020). Memristive circuit implementation of a self-repairing network based on biological astrocytes in robot application. IEEE Trans. Neural Networks Learn. Syst..

[5] Zhan Q., Liu G., Xie X., Zhang M., Sun G. (2023). Bio-inspired active learning method in spiking neural network. Knowl-Based Syst..

[6] Norton P., Benichov J.I., Pexirra M., Schreiber S., Vallentin D. (2022). A feedforward inhibitory premotor circuit for auditory–vocal interactions in zebra finches. Proc. Natl. Acad. Sci. USA.

[7] Rhodes O., Peres L., Rowley A.G., Gait A., Plana L.A., Brenninkmeijer C., Furber S.B. (2020). Real-time cortical simulation on neuromorphic hardware. Philos. Trans. R. Soc. A Math. Phys. Eng. Sci..

[8] Wu F., Ma J., Zhang G. (2020). Energy estimation and coupling synchronization between biophysical neurons. Sci. China Technol. Sci..

[9] Ward M., Rhodes O. (2022). Beyond LIF neurons on neuromorphic hardware. Front. Neurosci..

[10] Wei Y., McGlone F.P., Marshall A.G., Makdani A., Zou Z., Ren L., Wei G. (2022). From skin mechanics to tactile neural coding: Predicting afferent neural dynamics during active touch and perception. IEEE Trans. Biomed. Eng..

[11] Faci-Lázaro S., Soriano J., Mazo J.J., Gómez-Gardeñes J. (2023). Dynamical and topological conditions triggering the spontaneous activation of izhikevich neuronal networks. Chaos Soliton. Frac..

[12] Li Y., Fang L., Tao T., Li D., Liu E.X., Jin N., Ahmed M., Li E.P. (2022). Modeling and signal integrity analysis of RRAM-based neuromorphic chip crossbar array using partial equivalent element circuit (PEEC) method. IEEE Trans. Circuits Syst. I Regul. Pap..

[13] Yang W.C., Lin Y.C., Inagaki S., Shimizu H., Ercan E., Hsu L.C., Chueh C.C., Higashihara T., Chen W.C. (2022). Low-energy-consumption and electret-free photosynaptic transistor utilizing poly (3-hexylthiophene)-based conjugated block copolymers. Adv. Sci..

[14] Noh K., Cho W.H., Lee B.H., Kim D.W., Kim Y.S., Park K., Hwang M., Barcelon E., Cho Y.K., Lee C.J. (2023). Cortical astrocytes modulate dominance behavior in male mice by regulating synaptic excitatory and inhibitory balance. Nat. Neurosci..

[15] Sun X., Si H. (2020). Population rate coding in recurrent neuronal networks consisting of neurons with mixed excitatory–inhibitory synapses. Nonlinear Dyn..

[16] Taherkhani A., Belatreche A., Li Y., Cosma G., Maguire L.P., McGinnity T.M. (2020). A review of learning in biologically plausible spiking neural networks. Neural Netw..

[17] Zhang M., Wu J., Belatreche A., Pan Z., Xie X., Chua Y., Li G., Qu H., Li H. (2020). Supervised learning in spiking neural networks with synaptic delay-weight plasticity. Neurocomputing.

[18] Guo L., Yue H., Wu Y., Xu G. (2024). Complex spiking neural network with synaptic time delay evaluated by anti-damage capabilities under random attacks. Neurocomputing.

[19] van den Heuvel M.P., Stam C.J., Boersma M., Pol H.H. (2008). Small-world and scale-free organization of voxel-based resting-state functional connectivity in the human brain. Neuroimage.

[20] Budzinski R.C., Lopes S.R., Masoller C. (2021). Symbolic analysis of bursting dynamical regimes of Rulkov neural networks. Neurocomputing.

[21] Reis A.S., Brugnago E.L., Caldas I.L., Batista A.M., Iarosz K.C., Ferrari F.A., Viana R.L. (2021). Suppression of chaotic bursting synchronization in clustered scale-free networks by an external feedback signal. Chaos.

[22] Ardesch D.J., Scholtens L.H., Li L., Preuss T.M., Rilling J.K., van den Heuvel M.P. (2019). Evolutionary expansion of connectivity between multimodal association areas in the human brain compared with chimpanzees. Proc. Natl. Acad. Sci. USA.

[23] Li Y., Guan X., Yue W., Huang Y., Zhang B., Duan P. (2025). A Reinforced, Event-driven, and attention-based convolution spiking neural network for multivariate time series prediction. Biomimetics.

[24] Wu J., Xu C., Han X., Zhou D., Zhang M., Li H., Tan K.C. (2021). Progressive tandem learning for pattern recognition with deep spiking neural networks. IEEE Trans. Pattern Anal. Mach. Intell..

[25] Fiveash A., Bedoin N., Gordon R.L., Tillmann B. (2021). Processing rhythm in speech and music: Shared mechanisms and implications for developmental speech and language disorders. Neuropsychology.

[26] Tanaka G., Yamane T., Héroux J.B., Nakane R., Kanazawa N., Takeda S., Numata H., Nakano D., Hirose A. (2019). Recent advances in physical reservoir computing: A review. Neural Netw..

[27] Lu H., Lin X., Wang X., Du P. (2023). Spike-train level supervised learning algorithm based on bidirectional modification for liquid state machines. Appl. Intell..

[28] Zhang Y., Li P., Jin Y., Choe Y. (2015). A digital liquid state machine with biologically inspired learning and its application to speech recognition. IEEE Trans. Neural Networks Learn. Syst..

[29] Deckers L., Tsang I.J., Van Leekwijck W., Latré S. (2022). Extended liquid state machines for speech recognition. Front. Neurosci..

[30] Srinivasan G., Panda P., Roy K. (2018). Spilinc: Spiking liquid-ensemble computing for unsupervised speech and image recognition. Front. Neurosci..

[31] Guo L., Guo M., Wu Y., Xu G. (2023). Specific neural coding of fMRI spiking neural network based on time coding. Chaos Soliton. Frac..

[32] Song Y., Guo L., Man M., Wu Y. (2024). The spiking neural network based on fMRI for speech recognition. Pattern Recognit..

[33] Chen G., Kang B., Lindsey J., Druckmann S., Li N. (2021). Modularity and robustness of frontal cortical networks. Cell.

[34] Zhang Y., Sun H., Xie M., Feng Z., Wu Z. (2023). Enhancing Robustness of Memristor Crossbar-Based Spiking Neural Networks against Nonidealities: A Hybrid Approach for Neuromorphic Computing in Noisy Environments. Adv. Intell. Syst..

[35] Guo L., Song Y., Wu Y., Xu G. (2023). Anti-interference of a small-world spiking neural network against pulse noise. Appl. Intell..

[36] Jang J., Cho H., Kim J., Lee J., Yang S. (2020). Deep neural networks with a set of node-wise varying activation functions. Neural Netw..

[37] Guo L., Man R., Wu Y., Xu G. (2021). Anti-injury function of complex spiking neural networks under targeted attack. Neurocomputing.

[38] Qin K., Lei D., Pinaya W.H., Pan N., Li W., Zhu Z., Sweeney J.A., Mechelli A., Gong Q. (2022). Using graph convolutional network to characterize individuals with major depressive disorder across multiple imaging sites. EBioMedicine.

[39] Wu Y.J., Hou X., Peng C., Yu W., Oppenheim G.M., Thierry G., Zhang D. (2022). Rapid learning of a phonemic discrimination in the first hours of life. Nat. Hum. Behav..

[40] Liang J., Wang S.J., Zhou C. (2022). Less is more: Wiring-economical modular networks support self-sustained firing-economical neural avalanches for efficient processing. Natl. Sci. Rev..

[41] Peraza L.R., Taylor J.P., Kaiser M. (2015). Divergent brain functional network alterations in dementia with Lewy bodies and Alzheimer’s disease. Neurobiol. Aging.

[42] Li X., Yang C., Xie P., Han Y., Su R., Li Z., Liu Y. (2021). The diagnosis of amnestic mild cognitive impairment by combining the characteristics of brain functional network and support vector machine classifier. J. Neurosci. Methods.

[43] Ionescu T.M., Amend M., Hafiz R., Biswal B.B., Wehrl H.F., Herfert K., Pichler B.J. (2021). Elucidating the complementarity of resting-state networks derived from dynamic [18F] FDG and hemodynamic fluctuations using simultaneous small-animal PET/MRI. Neuroimage.

[44] Eguiluz V.M., Chialvo D.R., Cecchi G.A., Baliki M., Apkarian A.V. (2005). Scale-free brain functional networks. Phys. Rev. Lett..

[45] Destexhe A., Mainen Z., Sejnowski T.J. (1994). An E cient method for computing synaptic conductances based on a kinetic model of receptor binding. Neural Comput..

[46] Kleberg F.I., Fukai T., Gilson M. (2014). Excitatory and inhibitory STDP jointly tune feedforward neural circuits to selectively propagate correlated spiking activity. Front. Comput. Neurosci..

[47] Jiménez-Fernández A., Cerezuela-Escudero E., Miró-Amarante L., Domínguez-Morales M.J., de Asís Gómez-Rodríguez F., Linares-Barranco A., Jiménez-Moreno G. (2016). A binaural neuromorphic auditory sensor for FPGA: A spike signal processing approach. IEEE Trans. Neural Networks Learn. Syst..

[48] Petro B., Kasabov N., Kiss R.M. (2019). Selection and optimization of temporal spike encoding methods for spiking neural networks. IEEE Trans. Neural Networks Learn. Syst..

[49] Izhikevich E.M. (2003). Simple model of spiking neurons. IEEE Trans. Neural Networks.

[50] Wadatsumi T., Monta K., Hayashi Y., Miki T., Hatzopoulos A.A., Barić A., Nagata M. (2024). Chip-backside vulnerability to intentional electromagnetic interference in integrated circuits. IEEE Trans. Electromagn. Compat..

[51] Yang Y.T., Tien H.C., Chueh C.C., Lee W.Y. (2022). Polymer synaptic transistors from memory to neuromorphic computing. Mater. Chem. Phys..

[52] Barrat A., Barthélemy M., Vespignani A. (2004). Weighted evolving networks: Coupling topology and weight dynamics. Phys. Rev. Lett..

[53] Antoniou I.E., Tsompa E.T. (2008). Statistical analysis of weighted networks. Discrete Dyn. Nat. Soc..

[54] Yi C., Fan Y., Wu Y. (2023). Cross-module switching diversity of brain network nodes in resting and cognitive states. Cogn. Neurodyn..

[55] Vriend C., Wagenmakers M.J., van den Heuvel O.A., Van der Werf Y.D. (2020). Resting-state network topology and planning ability in healthy adults. Brain Struct. Funct..

